# Understanding Barriers to Healthcare Access for Heart Failure Patients during the COVID-19 Pandemic

**DOI:** 10.1093/geroni/igab046.3644

**Published:** 2021-12-17

**Authors:** Hanzhang Xu, Julie Miller, Roy Thompson, Bradi Granger, Matthew Dupre

**Affiliations:** 1 Duke University, Durham, North Carolina, United States; 2 Duke University School of Medicine, Duke University School of Medicine, North Carolina, United States; 3 Duke University, Duke University School of Nursing, North Carolina, United States

## Abstract

Early outpatient follow-up within two weeks after hospital discharge is an effective strategy for improving transitions of care in older patients with heart failure (HF). However, implementing timely follow-up care for HF patients has been challenging, especially during the COVID-19 pandemic. This convergent mixed-methods study identified patients’ barriers to accessing care and ascertained their recommendations for addressing these barriers. We enrolled 264 HF patients admitted to the Duke Heart Center between May 2020 and August 2021. A standardized survey and electronic health records (EHR) were used to collect patients’ sociodemographic, psychosocial, behavioral, and clinical data. For patients who reported some difficulty accessing their healthcare (n=30), semi-structured interviews were conducted to understand these barriers. Data were analyzed using rapid analysis techniques. Barriers to accessing care varied across participants, with scheduling an appointment being the most common barrier (12 of the 30 responses). Participants indicated that job-related conflicts, providers’ availability, or COVID-19 contributed most to the difficulty in scheduling an appointment. Some participants experienced more difficulties during the pandemic due to fewer appointments available for non-acute and non-COVID-19 related needs. Transportation was another critical barrier, which was often associated with the participants’ physical functional status. Participants identified the benefits of using telemedicine to address access to care barriers; however, they shared their concerns that telemedicine visits may not be sufficient to assess their HF conditions. Study findings highlight the need for more continual, tailored, and patient-centered interventions to improve access to care in older HF patients.

